# Modeling Age-Friendly Environment, Active Aging, and Social Connectedness in an Emerging Asian Economy

**DOI:** 10.1155/2016/2052380

**Published:** 2016-05-18

**Authors:** Ming-Ming Lai, Shi-Ying Lein, Siok-Hwa Lau, Ming-Ling Lai

**Affiliations:** ^1^Faculty of Management, Multimedia University, Persiaran Multimedia, 63100 Cyberjaya, Selangor, Malaysia; ^2^Faculty of Business, Multimedia University, Jalan Ayer Keroh Lama, 75450 Bukit Beruang, Melaka, Malaysia; ^3^Faculty of Accountancy, Universiti Teknologi MARA, Level 14, Menara SAAS, 40450 Shah Alam, Selangor, Malaysia

## Abstract

This paper empirically tested eight key features of WHO guidelines to age-friendly community by surveying 211 informal caregivers and 402 self-care adults (aged 45 to 85 and above) in Malaysia. We examined the associations of these eight features with active aging and social connectedness through exploratory and confirmatory factor analyses. A structural model with satisfactory goodness-of-fit indices (CMIN/df = 1.11, RMSEA = 0.02, NFI = 0.97, TLI = 1.00, CFI = 1.00, and GFI = 0.96) indicates that transportation and housing, community support and health services, and outdoor spaces and buildings are statistically significant in creating an age-friendly environment. We found a statistically significant positive relationship between an age-friendly environment and active aging. This relationship is mediated by social connectedness. The results indicate that built environments such as accessible public transportations and housing, affordable and accessible healthcare services, and elderly friendly outdoor spaces and buildings have to be put into place before social environment in building an age-friendly environment. Otherwise, the structural barriers would hinder social interactions for the aged. The removal of the environmental barriers and improved public transportation services provide short-term solutions to meet the varied and growing needs of the older population.

## 1. Introduction

Malaysia is the best place to retire in Asia and ranked third in the world according to the International Living Global Retirement Index 2014 [1]. Malaysia will be one of the aging nations by year 2030 with 15% of its population at the age of 60 and above [2]. Aging populations bring many implications that will increase the demand for long-term care rather than acute care. The elderly may have a range of disabilities, from performing daily activities to home management activities. Hence, it is imperative for Malaysia to have social policy reforms that cover areas from healthcare to building age-friendly communities for her aging society.

Effective policies that promote autonomy and enhance the quality of life among the elderly should be formed at the soonest [3]. An effective policy reformation should take into account age-friendly concepts introduced by the World Health Organization [4]. As stated by Menec et al. [5], despite WHO's [4] concept being appealing, “the research that has examined age-friendliness in diverse contexts is only starting to emerge” [5, page 15]. Prompted by these concerns, this paper is motivated to examine the associations between age-friendly environment, active aging, and social connectedness in addition to identifying the factors of aged-friendly features of the WHO [4] in Malaysia. A model with goodness-of-fit that uses structural equation modeling which integrated age-friendly features with active aging and social connectedness was built. Currently, this is a new attempt and is among the very few age-friendly environment studies. Such integration has not been investigated adequately especially in relation to the age-friendly environment, active aging, and social connectedness from the perception of middle-age adults and the above population. In particular, the significance of the eight age-friendly features of WHO [4] on active aging and social connectedness in a developing nation has yet to be fully understood in terms of the validity of a model with goodness-of-fit indices. Structural equation modeling has a higher accuracy in addressing the measurement error of variances in which the validity of the model is proven statistically through the goodness-of-fit indices [6]. This study also bridges the gaps in the current literature by examining age-friendliness with its outcomes such as social connectedness and active aging as suggested by Menec et al. [5].

The proposed structural model has contributed additional empirical evidence to the current literature for Lawton's person-environment fit [7] and Bronfenbrenner's ecological theory [8]. The findings of this study show in developing nations age-friendly domains relating to built environment where transportation and outdoor spaces and buildings need to be put in-place first before working on domains related to social interactions. It is because there are many infrastructural barriers in terms of transportation, housing, outdoor spaces, and buildings as perceived by the middle-age and older population. Accessibility and availability of modern public transportation are viewed as essential elements for social connectedness as well as participation in health and community services. An age-friendly environment “adapts its structures and services to be accessible to, and inclusive of older people with varying needs and capacities” [9, page 2].

## 2. Literature Review

Major studies related to aged-friendly environments, active aging, and social connectedness were reviewed. Feldman and Oberlink [10] are two of the pioneers in conceptualizing an age-friendly environment. By having 14 focus groups among individuals aged 35 and above and community leaders from various areas of the United States, they identified four domains of an age-friendly environment. The domains are identifying necessities, encouraging social and civic engagement, improving independence among the frail and disabled elderly, and optimizing physical and mental functioning and well-being.

The qualitative exploration of an age-friendly environment continued with Alley et al. [11] using the Delphi method to solicit opinions from 15 professionals in the areas of gerontology, township planning, and development. The results reveal that accessible and inexpensive transportation, housing, healthcare, safety, and prospective social involvement are the criteria for an age-friendly environment.

Subsequently, the concept of age-friendliness received global attention when WHO introduced the age-friendly cities guide [4], in which an age-friendly city “encourages active aging by optimizing opportunities for health, participation, and security in order to enhance quality of life” [4, page 1]. It is the result of an international-based study that examined the criteria of an age-friendly city by conducting focus group surveys in 33 cities involving 1,485 elderly, 250 elderly caregivers, and 515 service providers in the year 2005. The eight age-friendly environment features are outdoor spaces and buildings, transportation, housing, social participation, respect and social inclusion, civic participation and employment, communication and information, and, finally, community support and health services.

From the ecological perspective, it is important to acknowledge and integrate the social and physical environment. It is further asserted when Lui et al. [12] conducted a meta-analysis on 32 publications related to age-friendly environment and found the essentiality of the integration between physical and social environment, along with cooperation between policy makers and the elderly in building an age-friendly environment.

Emlet and Moceri [9] examined the importance of social connectedness in an age-friendly environment through focus group interviews with 23 respondents who were above 40 years of age in a community forum in the United States. The three themes of analysis were social reciprocity, meaningful interactions, and structural needs and barriers. The results reinforced the importance of social connectedness in an elder-friendly community.

Kadoya [13] explored the social interaction between the elderly and the community in Akita city, where there are a high number of elderly people in Japan. He found that living arrangement and mobility significantly affect social interactions. Those who have the ability to drive participated more in community services. He concluded that a community should not neglect social inclusion as a domain for an age-friendly environment. Menec et al. [5] conclude that the social environment is easier to address when compared to the physical environment especially in deserted areas.

The word “active aging” was first introduced by the WHO. It is defined as the “process of optimizing for health, participation and security in order to enhance quality of life as people age” [14, page 12]. The WHO proposed six determinants for active aging, namely, health and social services, behavioral determinant, personal determinant, physical environment, social determinant, and economic determinant. Active aging is perceived as the “desire and ability of older adults to integrate physical activity into daily routines” and “engagement in economic and socially productive activities” [15, page 736]. In essence, an age-friendly neighborhood design is found to be important in supporting active aging.

Bowling [16] explored active aging by surveying 337 elderly British men and women using questionnaires. The survey results revealed that the majority of the elderly described active aging as having good physical and mental health while maintaining substantial relationship with the society.

The measurement model of active aging was proposed by Paúl et al. [17]. They introduced a model that helps the elderly adjust to their disability, promote social participation, and be involved actively in a healthy lifestyle, which would improve their quality of life. An et al. [18] conducted a survey among elderly Koreans. They found that public exercise areas have a significant relationship with the elderly to be physically active which helps promote healthy and active aging.

On the other hand, social connectedness affects the elderly's health and well-being. Fratiglioni et al. [19] discovered that social engagements and participation that promote healthy and active aging are indicative of a good life among the elderly. In contrast, withdrawing from social activities may result in mental sickness. Social connectivity is viewed as a major involvement in social activities and communications that may affect the well-being of the older population [20].

Cramm et al. [21] investigated the relationship between social capital, social cohesion, and well-being among 945 elderly in Rotterdam. Their findings indicated that receiving social support and having interdependent neighborhoods were important to the elderly and would enhance their well-being.

Through in-depth personal interviews, Franke et al. [22] found that self-help strategies, social connectedness, and physical environment do promote physical activities among active older adults. The findings documented evidence from an individual perspective that active older adults used their self-help strategies to promote physical activities and reciprocally physical activities benefit them in determining their self-worth. New policies in addressing social connectedness and accessibilities towards social and physical community services are highly required in supporting the mobility and independence among the aging population.

**Figure 1:**
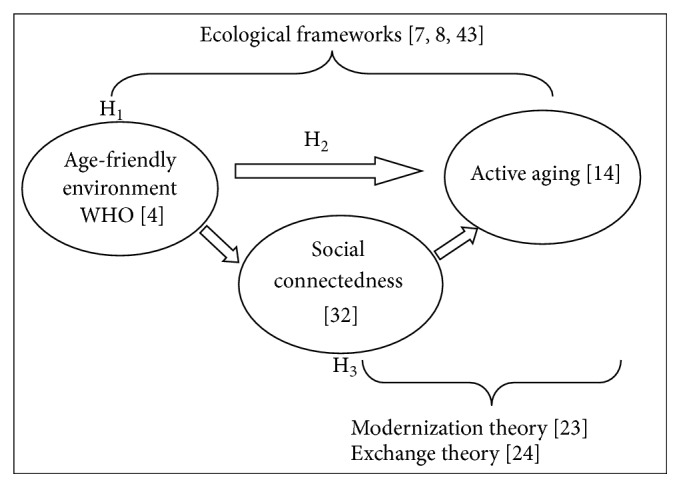
The integrated model of age-friendly environments, active aging, and social connectedness.

The literature review provides insights into an integrated model of an age-friendly environment, active aging, and social connectedness as shown in the theoretical framework (see Figure 1). It is believed that age-friendly environments and active aging have a substantive relationship and the relationship is mediated by social connectedness. Three hypotheses have been developed to examine the factors of age-friendly environments as well as the associations between age-friendly environments, active aging, and social connectedness. The proposed hypotheses are as follows:

H_1_: the factors (outdoor spaces and buildings, transportation, housing, social participation, respect, civic participation and employment, community support and health services, and communication and information) are positively related to age-friendly environments.
H_2_: an age-friendly environment is positively associated with active aging.
H_3_: social connectedness mediates the association between age-friendly environment and active aging.

First, the integrated model of age-friendly environments and active aging model serves as an attempt not only to improve the status of the elderly based on modernization [23] and the exchange of resources among the old-age population [24], but also to reduce environment pressure based on the environment docility hypothesis [7]. Social connectedness is found to be an important factor to be considered in building an age-friendly environment [9, 13] as well as promoting the well-being of the elderly [20, 21]. Motivated by these, social connectedness is perceived to mediate the direct relationship between age-friendly environment and active aging.

An age-friendly environment reflects the mesosystem of Bronfenbrenner's [8] ecological theory that addresses the interactivity of an individual with his or her immediate environment. This environment enables the elderly to be mobile, preserve their health, interact, and participate in their living community. The exosystem expands from the mesosystem and is reflected by the positive relationship between age-friendly environments and active aging. When the environment is friendly to the elderly, they are able to age actively and healthily. If the elderly are active and healthy in an age-friendly environment, their connectedness and participation in social activities would increase. Active aging receives support from social connectedness as indicated by the macrosystem of Bronfenbrenner's [8] ecological theory.

## 3. Data and Method

At the onset, a questionnaire was designed based on the criteria of urbanization level, old-age dependency ratio, and high number of the elderly people in Malaysia. The sample of this study is gathered from the states of Penang, Perak, Selangor, Wilayah Persekutuan Kuala Lumpur, Negeri Sembilan, Melaka, and Johor. The populations of interest are self-care adults and informal caregivers who do not have apparent cognitive impairment, aged 45 and above (or those in midlife or after midlife). Self-care adults are “people looking after themselves” [14, page 37]. They live in communities and not in institutions. Informal caregivers are individuals who provide care to the elderly.

Backman and Hentinen [25] defined self-care adults as those who have the “desire to continue living as an active agent” [25, page 567]. The targeted respondents are midlife and older adults. Jaques [26] argues that these adults have come to realize their own mortality and feel the need to reassess the changes they want to make while feeling that they still have the time. Individuals aged 45 to 64 are known as prepensioners [27]. The opinions and views from those who are not yet defined as older adults are helpful as they are able to tell of future needs that are beneficial for policy planning in an age-prepared community [9, 28]. Informed and written consent were sought before conducting the survey.

The questionnaire is in English language and translated into Malay and Chinese with the help of language experts. In order to ensure accuracy, an English language expert and researchers who are familiar with both these languages verified the Malay and Chinese versions of the questionnaires. Before the actual data collection, a pilot study was conducted among 30 adults aged 45 and above in December 2013. All constructs showed high reliability with Cronbach's alpha coefficient above 0.7.

All the items, except for demographic variables, were measured based on the five-point scale. Demographic variables encompass items such as gender, nationality, age, educational background, marital status, income level, employment status, and living arrangement. Since a common instrument to assess an age-friendly environment has yet to be established, it was self-developed by adapting the findings from an activity friendly community [29], the checklist of global age-friendly cities [30], and the Internet usage scale [31].

Age-friendly environment items evaluate the perception of the respondents at the level of importance of WHO's [4] age-friendly features in the community. They are measured by the 5-point scale ranging from 1 (very unimportant) to 5 (very important). Based on past literature, a commonly used active aging scale is yet to be found. Therefore, this study has developed its own active aging scale to measure the level of active aging that reflects quality of life and well-being. On the other hand, social connectedness is measured by adapting six items of the social connectedness of Lee and Robbins [32]. The social connectedness construct measures self-perceived emotional detachment or connectedness among individuals in a society. Higher scores indicate greater social connectedness, free from social isolation. Since the items are negatively worded, they are coded in reverse. Both the active aging and social connectedness scales were measured on the 5-point agreement Likert scale ranging from 1 (strongly disagree) to 5 (strongly agree).

In the earlier stage, both Cronbach's alpha reliability and exploratory factor analyses (EFA) were performed. Since the items in the constructs were yet to be established or well known, EFA was conducted to explore and validate the measurement items before moving onto the structural equation modeling (SEM) procedures. It is advisable to perform both the exploratory and confirmatory factor analyses by using new sample sets [33, 34]. Therefore, EFA was performed by employing the data from 211 informal caregivers and subsequently with confirmatory factor analysis (CFA) on 402 self-care adults. EFA was conducted on 37 items of the factors of age-friendly environment through the principal axis factoring (PAF) and promax rotation method. Items of the constructs used in SEM are attached in Appendix.

The hypothesized model is estimated based on a two-step structural equation modeling (SEM) approach, which starts with the specification of the measurement model. The values of skewness and kurtosis for each of the items are within the threshold of absolute two and seven, respectively, indicating that that data is normally distributed [35]. In addition, the maximum likelihood method is used to estimate the structural equation modeling. After confirming the fit of the measurement model, this study proceeded to the structural model. A model has a good fit when the goodness-of-fit indices are fulfilled. The goodness-of-fit indices are shown by an insignificant *p* value, observed normed chi-square value (CMIN/df) lower than 3, goodness-of-fit index (GFI), adjusted goodness-of-fit index (AGFI), comparative fit index (CFI), Tucker-Lewis Index (TLI), and normed fit index (NFI) to be more than 0.9, root mean squared error (RMSEA) and standardized root mean square residual (SRMR) less than 0.08, and expected cross validation index (ECVI) to be near zero [6, 36].

To examine the validity of the model, convergent validity and discriminant validity were performed. The assessment of convergent validity involves several indicators which are standardized factor loading and average variance extracted (AVE) to be at least 0.50 and composite reliability (CR) to be at least 0.70 [37]. AVE refers to the average of variance extracted of the items in each construct [38]. The formulas to calculate AVE and CR are shown below [37]:

(1) Average variance extracted=∑standardized factor loadings2Number of items;Composite reliability=∑standardized factor loadings2∑standardized factor loadings2+∑1−standardized factor loadings2.

Discriminant validity was measured by using the method formulated by Fornell and Larcker [38] whereby discriminant validity is achieved when AVE is larger than the value of the square of correlation between constructs [38]. The assessment of discriminant validity ensures that variables are unique and free from multicollinearity [37]. Variables with high correlation may be redundant and, hence, reduce validity. When the constructs in the measurement model have met all the fitness values, this study proceeded to perform the structural model.

The purpose of specifying a structural model is to conduct hypothesis testing. In testing the structural model, exogenous variables remained covariates from the measurement model but the double headed arrow of endogenous variables needed to be removed and replaced with a single headed arrow as well as adding a unique variable [37].

This study further examined social connectedness as a mediator in the hypothesized model. The steps for mediation were described as below [37]. First, independent and dependent variables need to have statistical significant relationships. Second, both the independent and dependent variables need to have statistical significant relationships with the mediating variable. When the mediating variable enters the model and causes the relationship between independent and dependent variables to become statistically insignificant, this result indicates a full mediating effect. On the other hand, when mediator variable enters the model, the relationship between independent and dependent variables remains statistically significant but with a reduced magnitude, this indicates a partial mediating effect. There is no mediating effect when the relationship between independent and dependent variables remains statistically significant and with similar coefficient when the mediating variable enters the model. In examining the mediation effect, resampling statistical procedure of bootstrapping was conducted by allowing data to perform 2,000 bootstrap samples and ran the bias-corrected confidence intervals at 95% [39, 40]. Sobel test was conducted to verify the mediation effect of social connectedness [41] and was calculated through a web-calculator developed by Preacher and Leonardelli [42].

## 4. Results

The usable sample size from the two surveys is 613 with 402 self-care adults and 211 informal caregivers. The results of the demographic characteristics of self-care adults and informal caregivers are reported in Table 1. The results show that 71.3% are female respondents, and the sample distributions are quite even across the seven states. The self-care adults of ages greater than 65 make up 47.3%. Meanwhile 49 informal caregivers are aged less than 35 (23.3%). About 25.9% of the respondents have primary school as their highest education level.

All the variables are reliable indicated by Cronbach's alpha greater than 0.7. Two items, each from housing and active aging variables, were removed to improve Cronbach's alpha value. The results of the reliability analysis are reported in Table 2.

The results of the exploratory factor analysis (EFA) show correlation of coefficients greater than 0.30, Kaiser-Meyer-Olkin (KMO) value at 0.92 (<0.60), a significant Bartlett test of sphericity (*p* = 0.00), and factor loadings greater than 0.40. Based on Kaiser's criterion, six factors were retained, but scree plot showed five factors to be retained. In combining Kaiser's criterion and scree plot test, only five factors were retained: communication and information, transportation and housing, outdoor spaces and buildings, community support and health services, and social participation and these five factors contributed to approximately 66% of the variances.

EFA showed only five factors, namely, (i) communication and information, (ii) outdoor spaces and buildings, (iii) housing and transportation, (iv) community support and health services, and (v) social participation, are significant in the Malaysian context. The results of EFA are presented in Table 3. These five factors were confirmed through CFA with the 402 self-care adults.

The initial measurement model had an unsatisfactory fit; therefore, a model modification was performed. In respecifying the measurement model, factor loadings, error terms, and modification indices were obtained to determine which items in the model should be dropped or added. This is to identify the best items of a construct before proceeding to specifying the structural model [36]. Modification indices of about four or larger indicate that model fit can be improved statistically by setting the estimated path to be free. They also indicate the possibility of cross-loadings among the problematic items [37].

During the model modification process, items with high modification indices were dropped after theoretical justification was employed. The two factors, namely, communication and information and social participation, were dropped. This is because, during the model respecification process, these two factors left with two items. Statistically, these two factors were underidentified and may be problematic in further SEM procedures [37]. The final measurement model has six constructs: outdoor spaces and buildings, transportation and housing, community support and health services, age-friendly environment, active aging, and social connectedness. The final measurement was adequately fit, indicated by *p* = 0.13, CMIN/df = 1.15, GFI = 0.96, RMSEA = 0.02, SRMR = 0.03, NFI = 0.97, TLI = 1.00, CFI = 1.00, AGFI = 0.95, and ECVI = 0.60.

The measurement model was examined using the convergent validity and discriminant validity tests. The result of convergent validity indicates that the variables achieved their convergent validity with average variance extracted greater than 0.50 as well as composite reliability greater than 0.70 (see Table 4). The results in Table 5 indicate that discriminant validity is attained when the values of average variance extracted in bold are all greater than the values of the square of correlations between variables that are not in bold.

**Figure 2:**
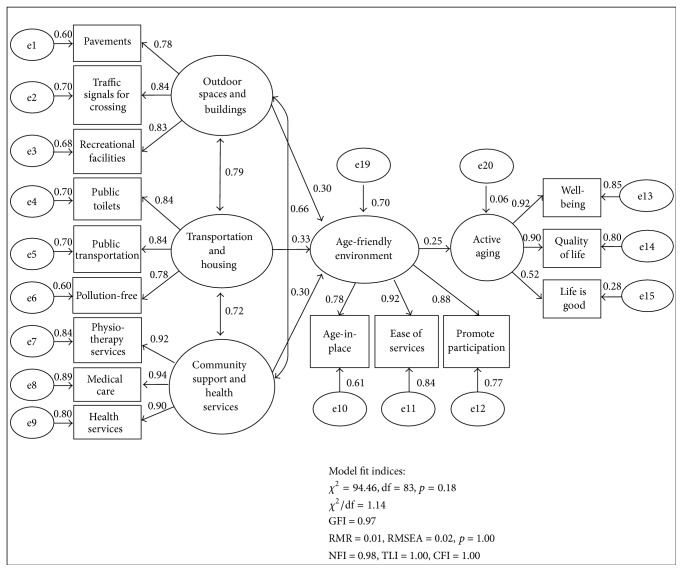
Structural model of age-friendly environment and active aging.

Table 6 reports the standardized coefficient of the variables in the structural model of age-friendly environment and active aging. The structural model has adequate goodness-of-fit; hence, the hypothesized model is considered reasonably fit. The model's goodness-of-fit indices have achieved the threshold with *p* = 0.18, CMIN/df = 1.14, GFI = 0.97, RMSEA = 0.02, SRMR = 0.02, NFI = 0.98, TLI = 1.00, CFI = 1.00, AGFI = 0.96, and ECVI = 0.42. The structural model is illustrated in Figure 2.

**Figure 3:**
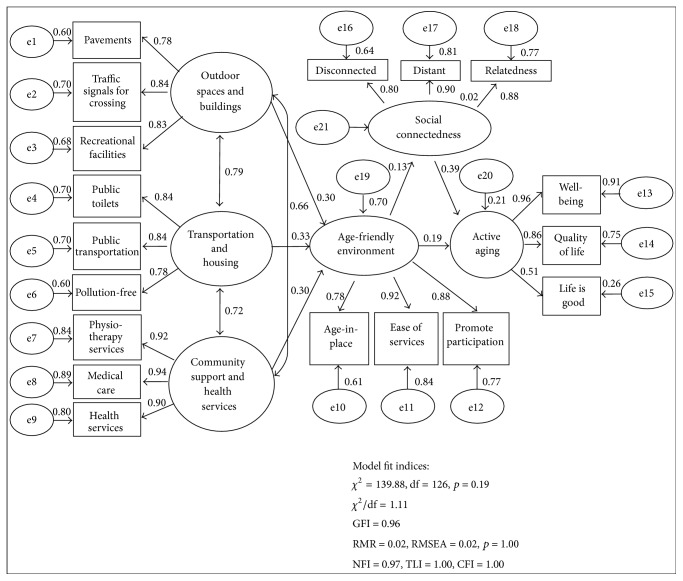
Overall structural model of age-friendly environment, active aging, and social connectedness.

The overall structural model with mediator is illustrated in Figure 3. The model fit values are within its threshold with *p* = 0.19, CMIN/df = 1.11, GFI = 0.96, RMSEA = 0.02, SRMR = 0.03, NFI = 0.97, TLI = 1.00, CFI = 1.00, AGFI = 0.95, and ECVI = 0.57. On the other hand, the results of mediation effect are reported in Table 7. The results verified the mediation model at 2.23 with *p* value of 0.03 with Sobel test [41] and through a web-calculator developed by Preacher and Leonardelli [42] (see Table 7).

The structural model shows that transportation and housing, outdoor spaces and buildings, and community support and health services represent 70.2% in total variance of an age-friendly environment. Transportation and housing factor is the most statistically significant factor in explaining an age-friendly model, followed by outdoor spaces and buildings and, lastly, community support and health services with standardized coefficient of 0.33, 0.30, and 0.30, respectively. All these three significant age-friendly features are found to be positively significantly associated with age-friendly environments. An age-friendly environment is positively significantly associated with active aging as shown by the standardized coefficient of 0.25 (*p* = 0.00), indicating that an age-friendly environment enables the elderly to age actively. Hence, H_1_ and H_2_ are supported.

The results show that an age-friendly environment is significantly linked to active aging and social connectedness with standardized coefficient of 0.25 and 0.13, respectively (see Table 7). Active aging is significantly related to social connectedness with standardized coefficient of 0.42. When social connectedness enters the model as a mediator, the relationship between an age-friendly environment and active aging remained statistically significant but at reduced standardized coefficient of 0.19. This is indicative of the fact that social connectedness partially mediates the relationship between an age-friendly environment and active aging, thus supporting H_3_. The standardized indirect effect of an age-friendly environment on active aging is statistically significant at 0.05.

## 5. Discussions and Implications

The overall structural model demonstrates a link between age-friendliness of the environment of an individual and active aging where the link is being partially mediated by social connectedness. The constructed structural model reflects the person-environment fit [7, 43] and is consistent with Bronfenbrenner's ecological theory [8]. Age-friendly environments enable adults to age actively. The results provide evidence that an age-friendly environment is significantly positively related to active aging [4, 12, 44]. Social participation with fair health promotes active aging [45]. The mediated model shows that 21% of active aging is explained by an age-friendly environment and social connectedness.

When adults are connected with society, emotional support received from their family and friends serves as emotional resources for them. Therefore, by increasing their social capital and gaining greater self-esteem and social status, they would be able to age actively. They further promote social connectedness that indirectly helps increase their exchange power and status in a modern society. Increase in exchange resources improves intergenerational relationships between them and their families as well as with their caregivers. Negative emotions for these older adults can be eliminated by reducing the imbalance in exchange relationships [46]. Harmonious familial relationships that support the emotional well-being of the older adults are formed.

It is surprising that not all of the eight main domains of the WHO [4] age-friendly features are perceived to be important by these respondents in Malaysia. Further discussions and analyses would provide insights in explaining the age-friendly environment from the Malaysian perspectives. First, the transportation and housing domain is perceived as the most important feature in building an age-friendly environment. The age-friendly transportation and housing would enable the elderly to participate in local community social events, exercise in parks, receive adequate healthcare, and visit family and friends, which would help promote social connectedness and participation [9]. Privately owned cars have become a common mode of transportation in Malaysia [47]. Given the fact that communities in Malaysia are highly car dependent, this has created challenges when older people are no longer able to drive. The unavailability and inaccessibility of the public transport system have indirectly affected other domains such as quality of social participation and civic engagement [13]. “The ability to drive (and feeling safe while driving) is crucial to social participation” [48, page 227].

This model helps policy makers and relevant authorities to understand that a public transportation system that is accessible, safe, and convenient helps to improve mobility when a person ages. This is what Malaysia should learn from developed countries [49]. Having accessibility to community services not only improves social and physical activities, but also provides an alternative to enhance the health of the elderly [22].

Second, outdoor spaces and buildings play an important role in the adaptability of adults in their living environment. Safe outdoor spaces equipped with the appropriate facilities help ease the mobility of adults, especially older adults [4]. The safe and elder-friendly outdoor spaces and buildings are in line with the person-environment fit [7, 43] which increases the level of adaptation and reduces the burden that arises from the environment. It further promotes the concept of aging-in-place where the elderly could continue to live in their community safely and independently [20].

In order for an individual to actively age and age-in-place, outdoor spaces and buildings that have friendly built environment are essential. The findings have contributed to the relevant authorities, especially urban planners by enlightening them on the importance of modifying physical design for outdoor spaces and buildings to be more elder-friendly to improve accessibility and walkability [50].

The community support and health services domain is perceived as another significant age-friendly feature. It serves as a proactive intervention in light of increasing healthcare cost resulting from poor health and chronic diseases among aging people. These findings are strongly in line with the need for a reform in the local healthcare system [51]. The main healthcare system in Malaysia concentrates predominantly on acute treatment, which may be inadequate to cater to the growing number of the elderly people, especially those with chronic illness and disabilities [52]. Healthy living can be promoted with the availability of public exercise areas in the communities [18]. This is also in line with the findings from the University of Hong Kong [53], where a practical community support system is included in an age-friendly environment. Given the limited physiotherapy facilities in the current communities in Malaysia and that fact that this service is essential for the recovery of the disabled elderly especially those suffering from stroke [54], we strongly suggest that the Malaysian government provides more public rehabilitation centers or physical exercise centers.

It is worth noting that the other four domains of the age-friendly features are perceived to be not significant in the Malaysian context. They are respect and social inclusion, social participation, civic participation and employment, and communication and information. Possible explanations are discussed below.

First, the lack of reliable and accessible transportation may have created barriers for the middle-aged and above respondents from participating in social, civic, and employment activities within their communities. As a result, they seldom have the opportunities to be included in social activities. It is perceived that limited interaction between the older and younger generations has indirectly led to the lack of respect of the younger generation towards older people.

Second, given that the age-friendly community concept is still very new in Malaysia, not all the outdoor spaces and buildings as well as working environments are elderly friendly. At the same time, modification plans to improve accessibility and walkability are limited. With limited working opportunities for the older people especially after retirement, it may hinder the elderly from being eligible for work and community participation [55].

Last but not least, high Internet connection cost as perceived by the respondents has indirectly discouraged them from accessing information and communication technologies. Some older respondents especially those with a lower education do not have the technical know-how to use advanced technologies, such as personal computers and smart phones. In view of this, we propose that lifelong learning classes should be organized for older adults by nongovernment organizations or local authorities.

Menec et al. [5] state that age-friendly domains related to social environment are easier to address than physical environment; however, this is not the case in the Malaysian environment context. As Malaysia is still a developing nation, many physical environments and infrastructures such as modern public transportation are yet to be fully developed. Hence, the findings strongly suggest that Malaysian policy makers need to prioritize the development of friendly built environments to reduce these structural barriers when building an age-friendly environment.

It should be pointed out that an enabling social environment is just as important as material conditions in determining the well-being for older people. As a whole, an age-friendly community should provide a comprehensive and accessible physical and social environment in which health, social involvement, and security of the elderly can be supported [4, 44].

The findings provide better understandings of an age-friendly environment which supports the well-being of the middle-agers and the elderly by enabling them to continue to be active in society as they age. The findings have potential to reduce social isolation among the aged. Nevertheless, the final model explains 70% of the age-friendly environment, 2% of social connectedness, and 21% of active aging. Hence, certain percentages of variables are yet to be explained especially the variable of social connectedness and active aging which would be of great interest for future studies.

## 6. Conclusions

Overall, the concept of age-friendly is still very new to Malaysia. Age-friendly transportation, outdoor spaces and buildings, and community and health services emerge as the three most important features in building an age-friendly environment. A structural model with satisfactory goodness-of-fit is built. Improved transportation services as well as modifications to outdoor spaces and buildings such as accessible public toilets and senior buses are essential as short-term solutions to reduce the structural barriers for the aged group to age actively and maintain connectedness to their community. Similarly, wide pavements and traffic signals for crossings as well as recreational activities are age-friendly features that can assist the adult groups in a physical environment. Not only that but also affordable medical care services and physiotherapy centers as well as convenient health services are identified as significant domains in building an age-friendly community. Domains related to social participation, respect and social inclusion, and civic engagement need more emphasis.

The findings provide better understandings for the state and municipal governments to implement age-friendly community initiatives to support the well-being of the residents by enabling them to continue to be active in society when they age. The findings have potential to reduce social isolation among the aged. Future research to examine the applicability and readiness of age-friendly features and outcomes in local communities would be of great interest. Last but not least, an age-friendly community index should be proposed to indicate the degree of age-friendliness.

## Figures and Tables

**Table 1:** Demographic characteristics of self-care adults and informal caregivers.

Demographic characteristics	Frequency, *n* (%)		
	*n* = 402^a^	*n* = 211^b^	*n* = 613^c^
Location (states)			
Pulau Pinang	57 (14.2)	28 (13.3)	85 (13.8)
Perak	50 (12.4)	30 (14.2)	80 (13.1)
Selangor	45 (11.2)	45 (21.3)	90 (14.6)
Wilayah Persekutuan Kuala Lumpur	77 (19.2)	17 (8.1)	94 (15.3)
Negeri Sembilan	58 (14.4)	30 (14.2)	88 (14.4)
Melaka	57 (14.2)	31 (14.7)	88 (14.4)
Johor	58 (14.4)	30 (14.2)	88 (14.4)
Gender			
Male	128 (31.8)	48 (22.7)	176 (28.7)
Female	274 (68.2)	163 (77.3)	437 (71.3)
Nationality			
Malaysian	402 (100)	211 (100)	613 (100)
Age			
≤35	—	49 (23.2)	49 (8.0)
35.1–45	—	37 (17.5)	37 (6.0)
45.1–55	66 (16.4)	44 (20.9)	110 (17.9)
55.1–65	146 (36.3)	45 (21.3)	191 (31.2)
≥65.1	190 (47.3)	36 (17.1)	226 (36.9)
Race			
Malay	141 (35.1)	50 (23.7)	191 (31.1)
Chinese	240 (59.7)	138 (65.4)	378 (61.7)
Indian	20 (5.0)	21 (10.0)	41 (6.7)
Portuguese	1 (0.2)	—	1 (0.2)
Orang Asli	—	2 (0.9)	2 (0.3)
Marital status			
Single	38 (9.5)	56 (26.5)	94 (15.3)
Married	260 (64.7)	142 (67.3)	402 (65.6)
Widowed	96 (23.9)	9 (4.3)	105 (17.1)
Divorced	8 (2.0)	4 (1.9)	12 (2.0)
Highest education level attained			
No formal education	47 (11.7)	13 (6.2)	60 (9.8)
Primary school	131 (32.6)	28 (13.3)	159 (25.9)
Secondary school	82 (20.4)	23 (10.9)	105 (17.1)
SPM^d^	65 (16.2)	37 (17.5)	102 (16.6)
STPM^e^/diploma	38 (9.5)	39 (18.5)	77 (12.6)
Degree	32 (8.0)	55 (26.1)	87 (14.2)
Postgraduate	7 (1.7)	16 (7.6)	23 (3.8)

*Total *	*402 (100)*	*211 (100)*	*613 (100)*

Notes: ^a^self-care adult respondents; ^b^informal caregiver respondents; ^c^self-care adult and informal caregiver respondents; ^d^Sijil Pelajaran Malaysia or also known as Malaysian Certificate of Education examination is equivalent to O-Level qualification; ^e^Sijil Tinggi Pelajaran Malaysia or also known as Malaysian Higher School Certificate examination which is equivalent to A-Level qualification.

**Table 2:** Reliability test results of self-care adults and informal caregivers.

Variables	Cronbach's alpha		
	*n* = 402^a^	*n* = 211^b^	*n* = 613^c^
Independent variables			
(1) Outdoor spaces and buildings	0.92	0.90	0.91
(2) Transportation	0.87	0.83	0.86
(3) Housing	0.91	0.89	0.90
(4) Social participation	0.84	0.89	0.86
(5) Respect and social inclusion	0.89	0.88	0.88
(6) Civic participation and employment	0.87	0.90	0.88
(7) Community support and health services	0.95	0.92	0.94
(8) Communication and information	0.98	0.96	0.97
Dependent variables			
(1) Age-friendly environment	0.95	0.95	0.95
(2) Active aging	0.86	0.85	0.85
(3) Social connectedness	0.95	0.94	0.95

Notes: ^a^self-care adult respondents; ^b^informal caregiver respondents; ^c^self-care adult and informal caregiver respondents.

**Table 3:** Exploratory factor analysis (EFA) on the factors of age-friendly environment employing the informal caregiver respondents.

Variables	Pattern matrix				
	1	2	3	4	5
(1) Communication and information					
(a) Stay in touch with people	0.97				
(b) Feel more connected	0.94				
(c) Easier to reach people	0.92				
(d) Increases communication	0.90				
(e) Feel less isolated	0.88				
(f) Easier to meet new people	0.74				
(2) Outdoor spaces and buildings					
(a) Good street lighting		0.91			
(b) Sufficient outdoor seating		0.84			
(c) Pavements are wide		0.81			
(d) Bicycle lanes and walking trails		0.78			
(e) Traffic signals for crossing		0.68			
(f) Recreational facilities		0.67			
(g) Interesting things to look at		0.49			
(3) Transportation and housing					
(a) Neighborhood safety			0.78		
(b) Drop-off and pickup areas			0.77		
(c) Accessible public transportation			0.73		
(d) Priority seating			0.71		
(e) Taxis are with discounts			0.62		
(f) Free of pollution			0.57		
(g) Available public transportation			0.56		
(h) Accessible public toilets			0.55		
(i) Walking trail from home to public transport station			0.55		
(4) Community support and health services					
(a) Reachable health services				0.88	
(b) Affordable medical care				0.86	
(c) Adequate range of services				0.72	
(d) Physiotherapy services				0.69	
(5) Social participation					
(a) Leisure activities					0.81
(b) Social activities					0.81
(c) Lifelong learning					0.81
(d) Religion activities					0.70
KMO	0.92				
Eigenvalue	13.96	4.09	2.14	1.77	1.21
Percentage of variance (%)	41.31	11.81	5.53	4.47	2.74

**Table 4:** Analysis of convergent validity of integrated model of age-friendly environment, active aging, and social connectedness.

Variables	Convergent validity			
	Standardized factor loadings	Average variance extracted	Composite reliability	Cronbach's alpha
(1) Outdoor spaces and buildings		0.66	0.86	0.85
(a) Pavements are wide	0.78			
(b) Traffic signals for crossing	0.84			
(c) Recreational facilities	0.83			
(2) Transportation and housing		0.67	0.86	0.86
(a) Accessible public toilets	0.84			
(b) Accessible public transportation	0.84			
(c) Free of pollution	0.78			
(3) Community support and health services		0.84	0.94	0.94
(a) Physiotherapy services	0.92			
(b) Affordable medical care	0.94			
(c) Reachable health services	0.90			
(4) Age-friendly environment		0.74	0.90	0.89
(a) Age-in-place	0.78			
(b) Ease of services to be delivered	0.92			
(c) Promote participation	0.88			
(5) Active aging		0.64	0.83	0.80
(a) Satisfied with well-being	0.96			
(b) Quality of life meets my expectation	0.86			
(c) Life is good	0.51			
(6) Social connectedness		0.74	0.90	0.89
(a) Disconnected from the world around me	0.80			
(b) Distant from people	0.90			
(c) Don't feel related to anyone	0.88			

Note: values of average variance extracted and composite reliability are calculated manually through the below formulas: average  variance  extracted = ∑standardized  factor  loadings^2^/number  of  items; composite  reliability = ∑(standardized  factor  loadings)^2^/(∑(standardized  factor  loadings)^2^ + (∑1 − standardized  factor  loadings^2^)).

**Table 5:** Analysis of discriminant validity of integrated model of age-friendly environment, active aging, and social connectedness.

Variables	Mean	SD	1	2	3	4	5	6
(1) Outdoor spaces and buildings	4.40	0.60	**0.66**					
(2) Transportation and housing	4.49	0.54	0.63 (0.79)	**0.67**				
(3) Community support and health services	4.50	0.56	0.43 (0.66)	0.52 (0.72)	**0.84**			
(4) Age-friendly environment	4.33	0.59	0.57 (0.76)	0.61 (0.78)	0.54 (0.74)	**0.74**		
(5) Active aging	3.82	0.67	0.03 (0.19)	0.04 (0.21)	0.02 (0.15)	0.06 (0.25)	**0.64**	
(6) Social connectedness	3.80	0.90	0.01 (0.10)	0.02 (0.12)	0.01 (0.11)	0.02 (0.12)	0.17 (0.42)	**0.74**

Notes: values in diagonal (bold) are average variance extracted (AVE) while values off-diagonal are the square of correlations between constructs; values in parentheses are correlations between constructs.

**Table 6:** Results of hypotheses testing.

Hypothesized path	*R* ^2^	Std. estimate^a^	CR^b^	Hypothesis supported
Variables	0.70			
Age-friendly environment				
Transportation and housing		0.33^*∗∗*^	4.04	Yes
Community support and health services		0.30^*∗∗*^	5.36	Yes
Outdoor spaces and buildings		0.30^*∗∗*^	4.03	Yes
Communication and information	Variable deleted			
Social participation	Variable deleted			
Civic participation and employment	Variable deleted			
Respect and social inclusion	Variable deleted			
Age-friendly environment → active aging	0.06	0.25^*∗∗*^	4.52	Yes

Notes: ^a^Std. estimate denotes standardized estimate; ^b^CR denotes critical ratio; ^*∗∗*^path is significant at *p* ≤ 0.01.

**Table 7:** Results of mediation effect.

Hypothesized path	Direct model without mediation	Standardized direct model with mediation	Standardized indirect model with mediation	CR^a^	Mediation effect	Hypothesis supported
Age-friendly environment → social connectedness	0.13^*∗*^	0.13^*∗*^	NA	2.34	NA	NA
Age-friendly environment → active aging	0.25^*∗∗*^	0.19^*∗∗*^	NA	3.89	NA	NA
Social connectedness → active aging	0.42^*∗∗*^	0.39^*∗∗*^	NA	7.52	NA	NA
Age-friendly environment → social connectedness → active aging	NA	NA	0.05^*∗∗*^	NA	Yes/partial	Yes

Notes: ^a^CR denotes critical ratio; NA denotes not applicable; ^*∗∗*^path is significant at *p* ≤ 0.01; ^*∗*^path is significant at *p* ≤ 0.05; Sobel test = 2.23 (*p* value = 0.03) calculated through a web-calculator developed by Preacher and Leonardelli [42].

## Appendix

### Items for the Constructs Used in SEM (in English Version)

The items of the constructs are as follows:

Age-friendly environment construct (1 = very unimportant, 2 = unimportant, 3 = neither unimportant nor important, 4 = important, and 5 = very important)

How important do you think each of these of age-friendly features in your community?

1. Outdoor spaces and buildings
  - Pavements are wide enough for elderly mobility
  - Presence of traffic signals for crossing with adequate time for elderly
  - Presence of recreational facilities
2. Transportation and housing
  - Public toilets are accessible conveniently
  - Public transportation is accessible by everyone including people with disabilities
  - Neighbourhood is free of pollution such as haze
3. Community support and health services
  - Affordable physiotherapy services are offered to restore elderly health
  - Affordable medical care services are available for elderly
  - Health services are conveniently reachable
4. Benefits of age-friendly community
  - Social environment promote age-in-place
  - Age-friendly environment make ease of services to be delivered to elderly
  - Age-friendly features promote participation that enhance elderly quality of life

Active aging construct (1 = strongly disagree, 2 = disagree, 3 = neither disagree nor agree, 4 = agree, and 5 = strongly agree)

Please indicate the response that best describes:

1. I am currently satisfied with my well-being
2. My quality of life meets my expectation
3. My life is good despite increased illness or physical disability.
